# Supplementary material for: The causal role of circulating inflammatory markers in osteoporosis: a bidirectional Mendelian randomized study
Source: Front Immunol. 2024 Jul 18;15:1412298. doi: 10.3389/fimmu.2024.1412298 (PMC11291241; doi:10.3389/fimmu.2024.1412298)
Supplement: Supplementary file 1 [file DataSheet_1.zip › supplementary material/OSTEOPOROSIS-CIM/GCST90274800.tsv.gz_forest.pdf]

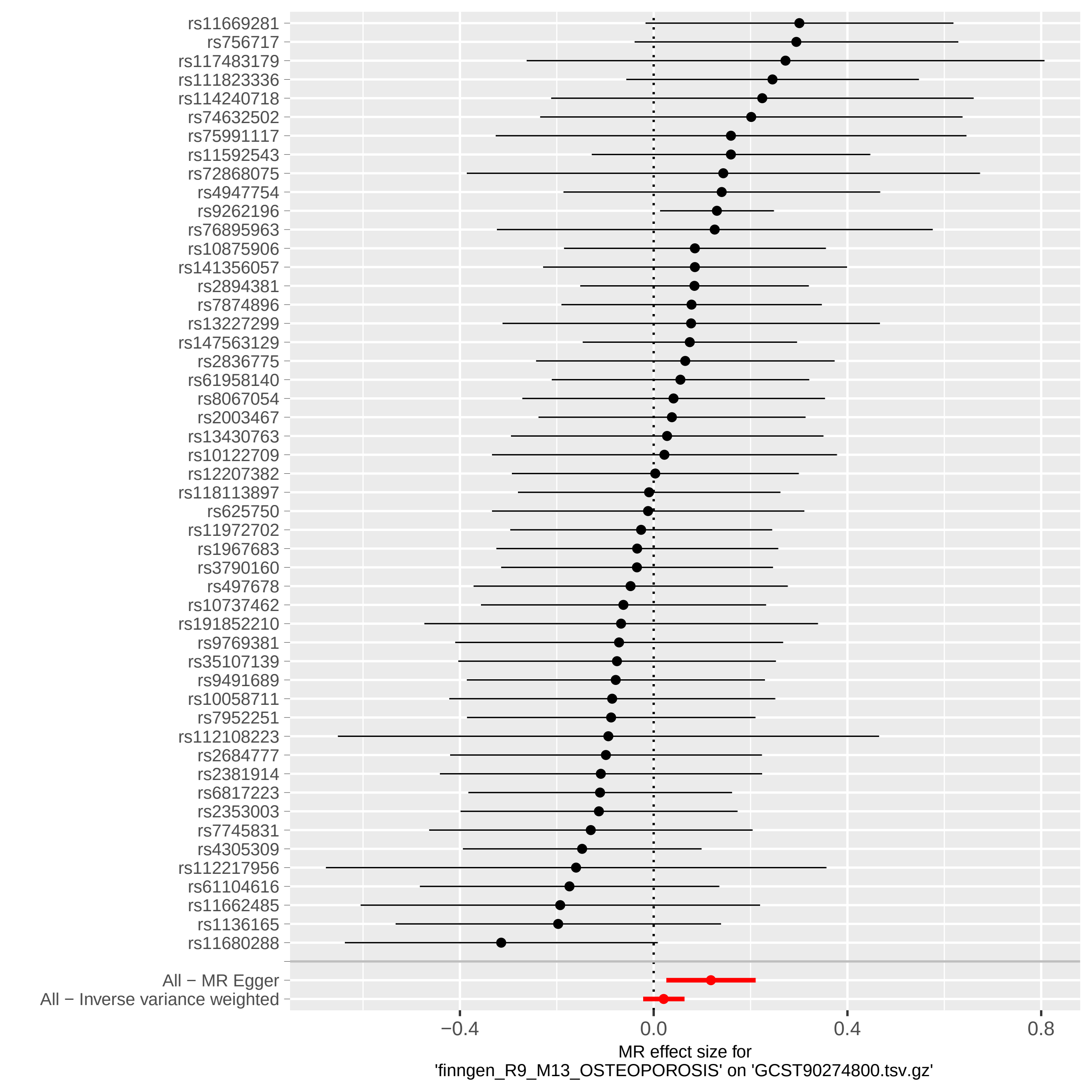
rs11669281
rs756717
rs117483179
rs111823336
rs114240718
rs74632502
rs75991117
rs11592543
rs72868075
rs4947754
rs9262196
rs76895963
rs10875906
rs141356057
rs2894381
rs7874896
rs13227299
rs147563129
rs2836775
rs61958140
rs8067054
rs2003467
rs13430763
rs10122709
rs12207382
rs118113897
rs625750
rs11972702
rs1967683
rs3790160
rs497678
rs10737462
rs191852210
rs9769381
rs35107139
rs9491689
rs10058711
rs7952251
rs112108223
rs2684777
rs2381914
rs6817223
rs2353003
rs7745831
rs4305309
rs112217956
rs61104616
rs11662485
rs1136165
rs11680288
All − MR Egger
All − Inverse variance weighted
−0.4
0.0
0.4
0.8
MR effect size for
'finngen_R9_M13_OSTEOPOROSIS' on 'GCST90274800.tsv.gz'
